# Characterization of bariatric surgery and outcomes using administrative claims data in the research network of a nationwide commercial health plan

**DOI:** 10.1186/s12913-021-06074-3

**Published:** 2021-02-04

**Authors:** Qinli Ma, Michael Mack, Sonali Shambhu, Kathleen McTigue, Kevin Haynes

**Affiliations:** 1 Translational Research for Affordability and Quality, HealthCore, Inc, Wilmington, DE USA; 2 Department of Epidemiology, University of Pittsburgh, Pittsburgh, PA USA

**Keywords:** Administrative claims data, Electronic health records data, Data integration, Bariatric surgery, PCORnet Clinical Research Networks

## Abstract

**Background:**

The supplementation of electronic health records data with administrative claims data may be used to capture outcome events more comprehensively in longitudinal observational studies. This study investigated the utility of administrative claims data to identify outcomes across health systems using a comparative effectiveness study of different types of bariatric surgery as a model.

**Methods:**

This observational cohort study identified patients who had bariatric surgery between 2007 and 2015 within the HealthCore Anthem Research Network (HCARN) database in the National Patient-Centered Clinical Research Network (PCORnet) common data model. Patients whose procedures were performed in a member facility affiliated with PCORnet Clinical Research Networks (CRNs) were selected. The outcomes included a 30-day composite adverse event (including venous thromboembolism, percutaneous/operative intervention, failure to discharge and death), and all-cause hospitalization, abdominal operation or intervention, and in-hospital death up to 5 years after the procedure. Outcomes were classified as occurring within or outside PCORnet CRN health systems using facility identifiers.

**Results:**

We identified 4899 patients who had bariatric surgery in one of the PCORnet CRN health systems. For 30-day composite adverse event, the inclusion of HCARN multi-site claims data marginally increased the incidence rate based only on HCARN single-site claims data for PCORnet CRNs from 3.9 to 4.2%. During the 5-year follow-up period, 56.8% of all-cause hospitalizations, 31.2% abdominal operations or interventions, and 32.3% of in-hospital deaths occurred outside PCORnet CRNs. Incidence rates (events per 100 patient-years) were significantly lower when based on claims from a single PCORnet CRN only compared to using claims from all health systems in the HCARN: all-cause hospitalization, 11.0 (95% Confidence Internal [CI]: 10.4, 11.6) to 25.3 (95% CI: 24.4, 26.3); abdominal operations or interventions, 4.2 (95% CI: 3.9, 4.6) to 6.1 (95% CI: 5.7, 6.6); in-hospital death, 0.2 (95% CI: 0.11, 0.27) to 0.3 (95% CI: 0.19, 0.38).

**Conclusions:**

Short-term inclusion of multi-site claims data only marginally increased the incidence rate computed from single-site claims data alone. Longer-term follow up captured a notable number of events outside of PCORnet CRNs. The findings suggest that supplementing claims data improves the outcome ascertainment in longitudinal observational comparative effectiveness studies.

**Supplementary Information:**

The online version contains supplementary material available at 10.1186/s12913-021-06074-3.

## Background

Electronic health records (EHR) data, with rich medical history and clinical information, is a real-world resource for conducting comparative effectiveness research [1]. However, most EHR systems keep medial encounters of a patient under a particular health care delivery system and are often challenged by incomplete data [2]. Reliance on EHR data from a single health system, however, could result in only partial capture of data across patients’ healthcare journeys, particularly when patients receive care from multiple health systems [3–7]. This presents challenges when studying long-term outcomes presenting greater concern for missing outcome events and biasing results.

For a more complete picture, a potential cost-efficient approach is to supplement EHR data with health insurance administrative claims data. Administrative claims data contains members’ health services information across health systems over defined enrollment periods. In fact, the use of claims data in comparative effectiveness studies is a reliable source of real-world data [8–11]. However, the value of supplementing claims data in ascertaining outcome events in longitudinal comparative effectiveness studies is not well quantified. One barrier is that integration of data from different sources with inconsistent quality exacerbates the difficulties of building complete longitudinal observational data sets [12–16].

Representing a new research paradigm, the Patient-Centered Outcomes Research Institute (PCORI) launched the National Patient-Centered Clinical Research Network (PCORnet) in 2014 in an effort to help address these challenges. The network leverages the power of health data and reusable research infrastructures from 13 Clinical Research Networks (CRNs) and two Health Plan Research Networks (HPRNs) to support multi-institutional research. Each CRN within PCORnet comprises health systems including hospitals, integrated delivery systems, and federally qualified health centers. Each HPRN is affiliated with a national health insurance provider. All CRNs and HPRNs store their EHR data and/or claims data in a common data format (known as PCORnet Common Data Model) that can be queried by researchers across institutions [17, 18]. Collectively, PCORnet includes more than 60 million geographically diverse patients, and is one of the largest and most representative healthcare research consortia in the United States.

One of the three PCORnet demonstration projects was the retrospective comparative effectiveness study on the three commonly performed types of weight loss surgery, adjustable gastric banding (AGB), Roux-en-Y gastric bypass (RYGB), and sleeve gastrectomy (SG) [19]. The study assessed the 5-year risks and benefits of bariatric surgical procedures in various populations [20–24]. This topic was selected as exemplary by PCORI firstly due to the increasing prevalence of obesity in the United States (with age-adjusted prevalence of 42.4% in adults in 2017–2018 [25]), and secondly due to the lack of evidence for SG. This procedure has less than a decade history in the country while it has quickly become the most commonly performed. The findings, particularly comparison of SG and bypass will enable patients and healthcare providers in making better-informed decision. The wide scope of data in PCORnet had the potential for large sample sizes that are essential to evaluate weight loss and rare adverse outcomes overtime [24].

Taking the PCORnet bariatric study as a model [22, 24], the objective of this study was to evaluate the utility of claims data in supplementing data available only to specific health systems (contained in EHR systems) in capturing outcomes longitudinally across health systems. We quantied the value of supplementing EHR data with claims data in outcome ascertainment in a large comparative effectiveness observational study with up-to 5 years follow-up. The claims data is from the HealthCore Anthem Research Network (HCARN), a participating PCORnet health plan that extracts administrative claims data from a large United States-based insurance company for inclusion in the PCORnet common data model. This study utilized claims data from HCARN to replicate the data a single health system (EHR data) would have available and compared outcome ascertainment limiting to data from a single health system to ascertainment utilizing complete claims profile across health systems.

## Methods

### Study design and data source

Eligible patients were identified via a computerized query for PCORnet bariatric computable phenotypes within the HCARN data stored in the PCORnet common data model (CDM). The HealthCore Intergraded Research Database (HIRD) is a repository of longitudinal claims data extracted, transformed, and loaded into the PCORnet CDM for HCARN enrollees, and is representative of the commercially insured population in the United States. Patient-level information for provider and facility visits in the HIRD were used to characterize care across healthcare delivery systems. HCARN members who had overlapping membership in a PCORnet CRN health system based on institutional claims for bariatric surgery were identified. Adverse events of interest for the overlapping patients in the claims data (based on outcome events in the PCORnet Bariatric Study [24]) were categorized as occurring either inside or outside PCORnet index CRN health systems. This study, which used a limited deidentified dataset, received an exemption from informed consent requirements by the New England Independent Review Board.

### Study population

The study population consisted of HCARN members who satisfied the criteria established for the PCORnet Bariatric Study using the 2016 version of the computable phenotype [12]. That is, patients who had one of the three common bariatric surgery procedures during inpatient or ambulatory care – AGB, RYGB or SG from 01/01/2007 to 09/30/2015 (see Additional file 1: Appendix Table 1 for International Classification of Diseases-9 (ICD-9) procedure codes and Current Procedural Terminology (CPT) codes used to identify bariatric procedures). The first procedure date was defined as the index date for each patient.

To be included, patients were required to be ≤79 years old at the index date. In addition, one year of continuous medical eligibility in a health plan prior to the index date was required for baseline evaluations. Patients with multiple conflicting bariatric procedure codes such as any revisional bariatric procedure code, gastrointestinal cancer diagnosis code, or fundoplasty in the year before the index procedure were excluded. Also excluded were members who had emergency department encounters on the day of the index bariatric procedure.

Post-exclusion, patients who had a bariatric surgery of interest at any PCORnet CRN health systems (based on institutional identifiers) were identified and included in the analysis. The health system where a patient had bariatric surgery was defined as the index CRN health system for that patient.

### Outcome events of interest

The main outcome measures, derived from the PCORnet Bariatric study, [12, 19, 22, 24] were safety outcomes requiring medical attention. The short-term outcome measure was a 30-day composite adverse event, including venous thromboembolism, percutaneous, operative, or endoscopic intervention, failure to discharge and death. The long-term outcome measures included major abdominal operation or intervention, all-cause hospitalization, and in-hospital death up to 5 years after bariatric surgery. For exploratory purposes, outcome measures that occurred within 1 year and 3 years post bariatric surgery were also examined to evaluate different follow-up times and the impact on outcome identification. In addition, death information was retrieved from the Social Security Administration, hospital discharge status in medical claims, and health plan enrollment file to evaluate overall mortality. Venous thromboembolism were identified by ICD-9 diagnosis codes, and abdominal operations/interventions were defined by ICD-9 procedure codes and CPT codes during a hospital stay. Patient follow-up was from the index date to the first of the following: end date of study, 09/30/2015; date of death, or to the cessation of health plan enrollment, and up to 1 year, 3 years and 5 years respectively.

Outcome events that occurred during the follow-up period were attributed to two exclusive categories in accordance with institutional identifiers linked to the hospital stay: within and outside the index CRN health system.

### Statistical analysis

Patients’ demographic characteristics, including age, gender, and region, were examined during the baseline period. Descriptive statistics were used to report outcome events that occurred within the index CRN health system to those that occurred outside CRN health systems. Incidence rates (per 100 patient-years) were reported respectively for 1) a single index health system, for which only the claims from a PCORnet health system were examined; 2) all health systems in the HCARN, for which full administrative claims data in HCARN were examined.

## Results

### Patient disposition

**Fig. 1:**
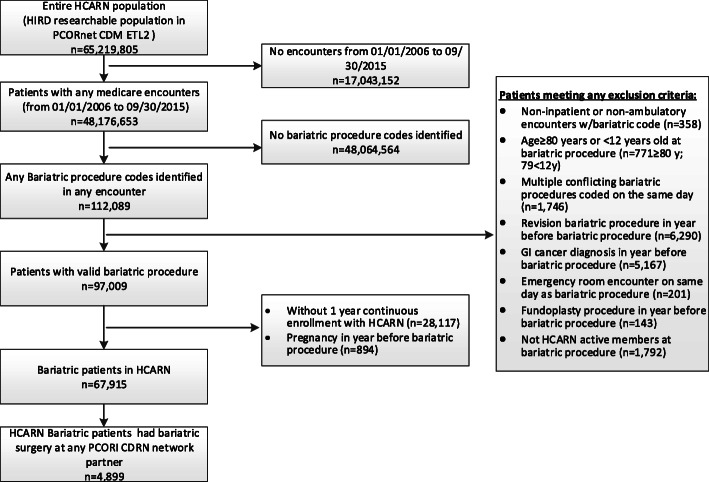
Patient attrition

A total of 112,089 bariatric surgery patients were identified in the overall HCARN cohort during the study period, as shown in Fig. 1. Upon applying age, enrollment, and clinical requirements, a total of 67,915 patients were selected. Among them, 4899 had an index bariatric surgery performed at a PCORnet CRN health system. Surgeries occurred in 66 health systems across 11 CRNs ranging from 27 to 1613 procedures (Additional file 1: Appendix Table 2).

The 4899 patients had a mean age of 44.6 years, was 70.5% female, and had average follow-up time of 28 months (median = 23 months). The Northeast and South regions each had slightly more than one third of the patients. The most commonly occurring comorbid conditions overall were hypertension (55.9%), diabetes mellitus (48.5%), dyslipidemia (43.1%), gastroesophageal reflux (31.1%), sleep apnea (26.4%), and depression (15.8%); mean (SD) Mixed Comorbidity Index score was 0.2 (1.13). About 34.3 and 39.4% patients received RYGB and AGB respectively, while 26.3% had SG procedures. From 2007 to 2015, a peak in the proportions of patients with AGB was observed in 2009 followed by a substantial decline afterwards. The proportion undergoing SG increased substantially and the trend for RYGB was relatively stable. The RYGB patients were slightly older, likelier to be female, and had more comorbid conditions, as shown in Table 1.

**Table 1:** Demographic and clinical characteristics at baseline

	All		AGB		RYGB		SG	
	n/mean	%/SD	n/mean	%/SD	n/mean	%/SD	n/mean	%/SD
**Number of patients, n (*N*** **= 4899)**	4899	100.0%	1681	34.3%	1932	39.4%	1286	26.3%
Age, mean (SD)	44.61	12.53	44.42	12.72	45.35	12.38	43.75	12.47
Age category, n (%)								
< 20	114	2.3%	33	2.0%	39	2.0%	42	3.3%
20–64	4561	93.1%	1567	93.2%	1792	92.8%	1202	93.5%
65–79	224	4.6%	81	4.8%	101	5.2%	42	3.3%
Female, n (%)	3456	70.5%	1152	68.5%	1394	72.2%	910	70.8%
Geographic region (Census Bureau), n (%)								
Northeast	1667	34.0%	684	40.7%	465	24.1%	518	40.3%
Midwest	929	19.0%	188	11.2%	551	28.5%	190	14.8%
West	677	13.8%	89	5.3%	306	15.8%	282	21.9%
South	1624	33.1%	719	42.8%	609	31.5%	296	23.0%
Medicare coverage, n (%)	175	3.6%	61	3.6%	88	4.6%	26	2.0%
Year of index surgery, n (%)								
2007	388	7.9%	230	13.7%	148	7.7%	10	0.8%
2008	442	9.0%	280	16.7%	151	7.8%	11	0.9%
2009	528	10.8%	331	19.7%	192	9.9%	<10	
2010	638	13.0%	326	19.4%	278	14.4%	34	2.6%
2011	718	14.7%	258	15.3%	323	16.7%	137	10.7%
2012	599	12.2%	104	6.2%	282	14.6%	213	16.6%
2013	575	11.7%	62	3.7%	232	12.0%	281	21.9%
2014	619	12.6%	57	3.4%	208	10.8%	354	27.5%
2015	392	8.0%	33	2.0%	118	6.1%	241	18.7%
Comorbid conditions,* n (%)								
Diabetes mellitus	2376	48.5%	814	48.4%	946	49.0%	616	47.9%
Gastroesophageal reflux	1525	31.1%	444	26.4%	699	36.2%	382	29.7%
Hypertension	2737	55.9%	898	53.4%	1147	59.4%	692	53.8%
Sleep apnea	1291	26.4%	275	16.4%	593	30.7%	423	32.9%
Deep vein thromboembolism	56	1.1%	19	1.1%	18	0.9%	19	1.5%
Pulmonary embolism	55	1.1%	21	1.2%	17	0.9%	17	1.3%
Dyslipidemia	2111	43.1%	742	44.1%	862	44.6%	507	39.4%
Anxiety	456	9.3%	106	6.3%	204	10.6%	146	11.4%
Depression	776	15.8%	189	11.2%	367	19.0%	220	17.1%
Eating disorder	137	2.8%	27	1.6%	55	2.8%	55	4.3%
Kidney disease	122	2.5%	30	1.8%	50	2.6%	42	3.3%
Non-alcoholic fatty liver disease.	193	3.9%	35	2.1%	89	4.6%	69	5.4%
Osteoarthritis	578	11.8%	198	11.8%	236	12.2%	144	11.2%
Polycystic ovarian syndrome	149	3.0%	43	2.6%	60	3.1%	46	3.6%
Psychotic disorder	433	8.8%	108	6.4%	196	10.1%	129	10.0%
Substance use disorder	31	0.6%	<10		17	0.9%	<10	
Mixed Comorbidity Index score, mean (SD)	0.2	1.13	0.08	1.07	0.28	1.21	0.23	1.07
Mixed Comorbidity Index score, n (%)								
< 0	1379	28.1%	536	31.9%	521	27.0%	322	25.0%
0	1997	40.8%	702	41.8%	750	38.8%	545	42.4%
> 0	1523	31.1%	443	26.4%	661	34.2%	419	32.6%
Hospital length of stay in days, mean (SD)	0.73	4.31	0.39	2.14	1.14	6.03	0.58	3.15

*Adapted from Flum et al. [26]

*RYGB* Roux-en-Y gastric bypass procedure, *AGB* Adjustable gastric banding, *SG* Sleeve gastrectomy

### 30-day composite adverse events

Among 4899 patients, 4752 continuously enrolled with their health plan for the 30 days after the index date, and therefore were included in the analysis of 30-day adverse events. Using HCARN multi-site claims data marginally impacted the incidence rate of using HCARN single-site claims data for index CRN health system, an increase from 3.9 to 4.2%, as shown in Table 2. A large majority of patients, 162 of 174 (93.1%), had their percutaneous or endoscopic intervention performed at the hospital where they received their index bariatric procedure, however, only 15 out of 19 (78.9%) had their venous thromboembolism treated at the index health system.

**Table 2:** Incidence rates for 30-day composite adverse events

Outcome Events (***n*** = 4752)	Index Health System		All health systems in HCARN	
**30d Composite Adverse Events**	183	3.85%	198	4.17%
Death	10	0.21%	11	0.23%
Venous thromboembolism	15	0.32%	19	0.40%
Percutaneous, operative, or endoscopic intervention	162	3.41%	174	3.66%
Failure to discharge from hospital within 30 days	19	0.40%	19	0.40%

*PCORnet* National Patient-Centered Clinical Research Network, *CRN* Clinical Research Networks, *HCAR*N HealthCore Anthem Research Network

In an exploratory analysis, we examined the 15 patients who were admitted to non-CRN hospitals for adverse events within 30 days. These 15 patients on average lived further away from the index CRN health system (average 55 miles, range from 6 miles to 142 miles at the zip code level) than from the hospital which treated the adverse outcome (average of 16 miles, range from 4 to 36 miles at zip code level).

### Long-term outcome measures

Across health systems using all available HCAR N claims, slightly more than a quarter of the patients, 1361 (27.8%), were admitted to hospitals, 540 (11.0%) had abdominal operations/interventions, and 31 (0.6%) died while in hospital up to 5 years after the bariatric surgery, as shown in Table 3. Upon the inclusion of all adverse events during the follow-up period, allowing for multiple admissions per patient, for the 4899 patients, there were 2893 hospital admissions, among which 701 were for abdominal operations or interventions.

**Table 3:** Adverse events up to 5 years follow-up

Outcome Events	Overall		Within index Health System		Outside index Health System	
**Number of patients**	4899					
**Follow up time, mean (median) in months**	28.0	23.2				
***All-cause hospitalization***						
≥ 1 hospital admission, n (%)	1361	27.8%	761	15.5%	780	15.9%
Number of visits, total (row %)	2893	100%	1250	43.2%	1643	56.8%
***a) within 1 year***						
≥ 1 hospital admission, n (%)	705	14.4%	442	9.0%	313	6.4%
Number of visits, total (row %)	1076	100%	593	55.1%	483	44.9%
***b) within 3 years***						
≥ 1 hospital admission, n (%)	1209	24.7%	687	14.0%	662	13.5%
Number of visits, total (row %)	2359	100%	1079	45.7%	1280	54.3%
***Abdominal operations/interventions***						
≥ 1 hospital admission, n (%)	540	11.0%	392	8.0%	175	3.6%
Number of visits, total (row %)	701	100%	482	68.8%	219	31.2%
***a) within 1 year***						
≥ 1 hospital admission, n (%)	500	10.5%	368	7.7%	154	3.2%
Number of visits, total (row %)	642	100%	449	69.9%	193	30.1%
***b) within 3 years***						
≥ 1 hospital admission, n (%)	518	10.9%	381	8.0%	162	3.4%
Number of visits, total (row %)	675	100%	470	69.6%	205	30.4%
***In-hospital Death***						
Number of death, total (row %)	31	100%	21	67.7%	10	32.3%

**Fig. 2:**
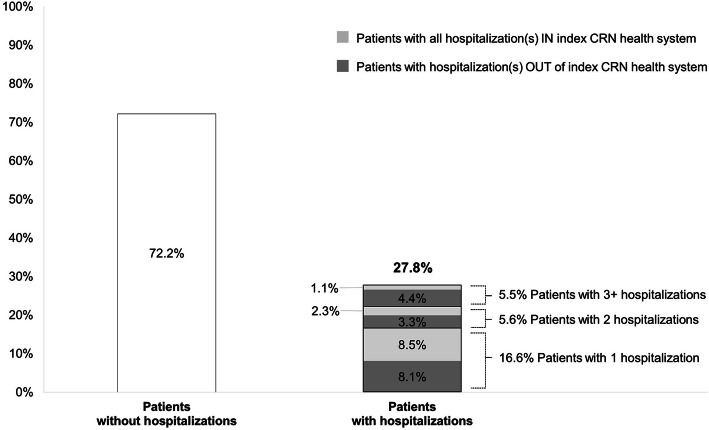
Individual patients by hospitalizations over follow up period

When examining individuals, 3538 (72.2%) patients had no hospitalization over the 5-year follow-up period. For those with hospital admission, 810 (16.5%) had 1 hospitalization in which 415 (51.2%) were admitted to the index CRN health system, that is, where the initial bariatric surgery was performed; 277 (5.6%) patients had two hospitalizations, in which 111 (40.2%) had both admissions in index health system and 60 (21.7%) had one admission in the index health system. Among 275 patients who had three or more hospital admissions, 55 (20.0%) had all admissions in index health system and 120 (43.6%) had at least one admission in index health system. Overall, 761 out of 1361 (55.9%) who had hospitalizations were admitted to the index CRN health system, and the other 600 patients did not return to the index health system during the follow-up (Fig. 2).

For hospital episodes, a total of 1250 or 43.2% of the 2893 hospitalizations occurred in the index CRN health system (Table 3), and 56.8% were admitted to hospitals outside of the index CRN health system. Hospital admissions to the index CRN health system were slightly more likely to occur right after the index surgery, 55.1% within 1 year and 45.7% within 3 years after index date. For the 701 hospitalizations for abdominal operations/interventions, 482 (68.8%) occurred in the index CRN health system, and 219 (31.2%) were outside of the index CRN health system. 91.6% (642 out of 701) abdominal operations/interventions were performed within 1 year after index surgery and the likelihood of occurrence in index health system was relatively stable over time.

An overall mortality rate of 1.4% (67 deaths out of 4899 patients) was observed using health plan claims data, health plan enrollment data, and Social Security Administration data. Of those, 46.3% (31 deaths) occurred in hospitals based on hospital discharge billing records in claims. For the 31 in-hospital deaths, 21 (67.7%) occurred at the index CRN health system, in which 8 occurred in the admission for the index bariatric surgery; and 10 (32.3%) outside of index CRN health system.

### Incidence rates

**Fig. 3:**
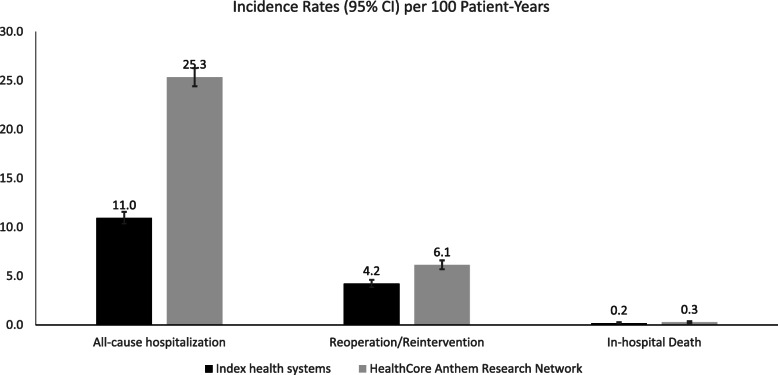
Incidence rates of long-term adverse events

The incidence rates increased when the claims data was expanded from a single index CRN health system to the full HCARN claims from across health systems, as shown in Fig. 3: all-cause hospitalization, 11.0 (95% Confidence Internal [CI]: 10.4, 11.6) to 25.3 (95% [CI]: 24.4, 26.3) visits per 100 patient-years; abdominal operations/interventions, 4.2 (95% [CI]: 3.9, 4.6) to 6.1 (95% [CI]: 5.7, 6.6) visits per 100 patient-years; in-hospital death, 0.2 (95% [CI]: 0.11, 0.27) to 0.3 (95% [CI]: 0.19, 0.38) death per 100 patient-years.

## Discussion

This observational study demonstrated the value of administrative claims data in enhancing the ability to follow patient outcomes across health systems longitudinally. In our study of patients undergoing the most common bariatric procedures, about 54% of subsequent all-cause hospitalizations, 31% abdominal operations or interventions, and 32% of in-hospital deaths occurred outside of the index CRN health system during the 5-year follow-up period. These events could be missed when data are only available from a single health system.

The estimated 30-day rate of composite adverse events based on the available administrative claims data in our study aligned well with prior work. In the PCORnet bariatric study, Arterburn et al. reported that 3.8% of their patients had composite adverse events within 30 days after bariatric surgery based on EHR data from PCORnet CRNs [22]. In our study, we observed rates of 3.9% using data from PCORnet CRN health systems and 4.2% using available data from HCARN, which were comparable to the Longitudinal Assessment of Bariatric Surgery (LABS) Study (4.1% for composite averse events) [26].

The addition of claims data to outcome events was highly related to the timing of the event. The closer an event was to the index date, the likelier it occurred at an index CRN health system. For adverse events within 30 days, 183 of 198 (92.4%) patients visited their index health system. For all-cause hospitalizations, the rate of index CRN health system admissions decreased from 55.1% in 1-year follow-up to 45.7% in 3-year follow-up. Abdominal operations or interventions in this study mainly occurred within 1 year after the index bariatric surgery, therefore, over time, approximately 69% of these procedures were at index CRN health systems.

Besides timing, the likelihood of receiving care at non-CRN health systems was related to the urgency of the event. For composite adverse events within 30 days, we observed that venous thromboembolism was more likely to occur outside of index CRN hospitals compared with surgical intervention (21.1% of overall venous thromboembolism and 6.9% of overall surgical interventions occurred outside of index hospital). Patients with pulmonary embolism or deep vein thrombosis require urgent care and would be directed to the closest emergency room for immediate treatment. In contrast, if patients require a reintervention of a prior bariatric surgery, they might be more inclined to return to the health system where they had their initial surgery. In addition, our exploratory analysis that showed longer travel distance from patient’s house to index hospital than to non-CRN hospital implied that location convenience of a health system played a role where patients are seeking urgent care.

One of the advantages of administrative claims data is the accuracy in measuring the enrollment time with informative censoring at time of disenrollment. Claims data have more accurate information on member censoring based on health plan enrollment records compared to EHR data, which often have incomplete information on when someone leaves a health system. This complete data capture of medically relevant events across health systems over defined periods of health plan enrollment is a strength of health plan claims data in longitudinal comparative effectiveness research.

Our results, on their own, and in conjunction with the findings of prior research that integrated clinical and claims data, have important implications for how treatment outcomes are tracked and managed across health systems. The value of claims data was demonstrated in our earlier PCORnet prospective longitudinal pragmatic study: Aspirin Dosing: A Patient-centric Trial Assessing Benefits and Long-Term Effectiveness (ADAPTABLE) trial designed to compare the effectiveness of two doses of aspirin. Applying the design of the ADAPTABLE trial in HCARN claims data, we found that claims data captured > 30% non-fatal and fatal hospital events in cardiovascular health occurring in health systems outside of a potential enrolling CRN health system [27]. Expansion of this approach could substantially increase the availability of information for clinical comparative effectiveness research.

Of note, the combined EHR and claims data is a rare but emerging resource in the United States outside of integrated delivery systems. Data integration is difficult due to fragmented healthcare delivery. There are additional challenges on data standardization and governance. PCORnet has worked to close these gaps through application of data standardization and proposed data linkage governance. Our findings support the need for continued work to integrate data sources for complete capture of clinical outcomes in observational analyses. Our research demonstrates the increase in identifying outcomes over longer follow-up periods closing significant data gaps and thus improving real-world evidence generation.

### Limitations

Administrative claims data lack clinical information, such as changes in weight, blood pressure, cholesterol, and diabetic glycemic control. Claims data is limited to data capturing medically attended events and data necessary to document coded billable encounters through the utilization of diagnosis, procedure, and medication dispensing codes. Our ability to accurately ascribe events to particular CRN health systems was limited on the completeness of tax identification numbers and linking their affiliations within known health systems. Administrative claims data may have coding inaccuracies, which might have introduced outcome misclassification into our study. In addition, patients included in this study were commercially insured and received care at PCORnet CRNs, consisting mainly of large academic medical centers in certain regions. Patients who seek care at these centers only reflect the utilization patterns in such institutions or geographic regions. Caution should be exercised when generalizing these results to different institutions, areas with less healthcare resources, or populations with different socioeconomic status.

## Conclusions

Our study showed that patients receive care across health systems depending on the nature of the event such as timing, emergency, or location convenience. Administrative claims data provide a system for longitudinal ascertainment of health outcomes across health systems over defined enrollment periods within a health plan. These results suggest that supplementing EHR data with administrative claims data will capture a fuller picture of outcome events in longitudinal observational comparative effectiveness studies.

## Supplementary Information

**Additional file 1.**

## Data Availability

The datasets generated and analyzed during the current study are not publicly available due to containing information that could potentially identify study participants, but are available from the corresponding author on reasonable request.

## Abbreviations

PCORI: Patient-Centered Outcomes Research Institute
PCORnet: Patient-Centered Clinical Research Network
HCARN: HealthCore Anthem Research Network
CRNs: Clinical Research Networks
HPRNs: Health Plan Research Networks
EHR: Electronic health records
AGB: Adjustable gastric banding
RYGB: Roux-en-Y gastric bypass
SG: Sleeve gastrectomy
CDM: Common data model
HIRD: HealthCore Intergraded Research Database
ICD: International Classification of Diseases
LABS: The Longitudinal Assessment of Bariatric Surgery Study
ADAPTABLE: Aspirin Dosing: A Patient-centric Trial Assessing Benefits and Long-Term Effectiveness
