# Supplementary material for: Characterization of bariatric surgery and outcomes using administrative claims data in the research network of a nationwide commercial health plan
Source: BMC Health Serv Res. 2021 Feb 4;21:116. doi: 10.1186/s12913-021-06074-3 (PMC7860025; doi:10.1186/s12913-021-06074-3)
Supplement: Supplementary file 1 — Additional file 1. [file 12913_2021_6074_MOESM1_ESM.docx]

**Appendix Tables**

**Table 1: ICD-9 and CPT-4 codes used to identify bariatric procedures**

| **Code** | **Description** | **Procedure Assignment** | **Code type** |
| --- | --- | --- | --- |
| 43.82 | Laparoscopic vertical (sleeve) gastrectomy | SG | ICD-9 |
| 43.89 | Partial gastrectomy with bypass gastrogastrostomy; Sleeve resection of stomach | SG | ICD-9 |
| 43775 | Laparoscopic sleeve gastrectomy | SG | CPT-4 |
| 44.31 | High gastric bypass; Printen and Mason gastric bypass | RYGB | ICD-9 |
| 44.39 | Other gastroenterostomy; Bypass gastroduodenostomy; gastroenterostomy; gastrogastrostomy; Gastrojejunostomy without gastrectomy NOS | RYGB | ICD-9 |
| 43633 | Gastrectomy, partial, distal; with Roux-en-Y reconstruction | RYGB | CPT-4 |
| 43846 | Gastric restrictive procedure, with gastric bypass, for morbid obesity; with short limb (less than 100 cm) Roux-en-Y gastroenterostomy | RYGB | CPT-4 |
| 43847 | Gastric restrictive procedure, with small intestine reconstruction to limit absorption; with long limb (>150 cm) Roux-en-Y | RYGB | CPT-4 |
| 44.38 | Laparoscopic gastroenterostomy; Bypass: gastroduodenostomy; gastroenterostomy; gastrogastrostomy; Laparoscopic gastrojejunostomy without gastrectomy NEC | RYGB | ICD-9 |
| 43644 | Laparoscopy, surgical, gastric restrictive procedure with gastric bypass and Roux-en-Y gastroenterostomy (roux limb 150 cm or less) | RYGB | CPT-4 |
| 43645 | Laparoscopy, surgical, gastric restrictive with gastric bypass and small intestine reconstruction to limit absorption | RYGB | CPT-4 |
| 43844 | Laparoscopic gastric restrictive procedure with gastric bypass and Roux-en-Y gastroenterostomy | RYGB | CPT-4 |
| S2085 | Lap GASTRIC BYPASS | RYGB | HCPCS |
| 44.95 | Laparoscopic gastric restrictive procedure | AGB | ICD-9 |
|  | Adjustable gastric band and port insertion |  |  |
| 43770 | Laparoscopy, surgical, gastric restrictive procedure: placement of adjustable gastric band | AGB | CPT-4 |
| S2082 | Lap Band | AGB | HCPCS |

AGB=adjustable gastric banding; CPT-4=Current Procedural Terminology, 4^th^ Edition, ICD-9=International Classification of Diseases, Ninth Revision; NEC=not elsewhere classifiable; NOS=not otherwise specified; RYGB=Roux-en-y gastric bypass procedure; SG=sleeve gastrectomy

**Table 2: Patients by PCORnet CRNs**

|  | **AGB** | **RYGB** | **SG** | **Total** |
| --- | --- | --- | --- | --- |
| Total | 1681 | 1932 | 1286 | 4899 |
| CRN 1 | 20 | 70 | 81 | 171 |
| CRN 2 | 20 | 147 | 124 | 291 |
| CRN 3 | 727 | 598 | 288 | 1613 |
| CRN 4 | 172 | 447 | 96 | 715 |
| CRN 5 | 8 | 6 | 17 | 31 |
| CRN 6 | 0 | 9 | 18 | 27 |
| CRN 7 | 652 | 331 | 444 | 1427 |
| CRN 8 | 11 | 24 | 42 | 77 |
| CRN 9 | 33 | 131 | 59 | 223 |
| CRN 10 | 9 | 93 | 56 | 158 |
| CRN 11 | 29 | 76 | 61 | 166 |

AGB = Adjustable gastric banding

RYGB = Roux-en-y gastric bypass procedure

SG = Sleeve gastrectomy

CRN = Clinical Research Networks
